# Effects of Pore Defects on Stress Concentration of Laser Melting Deposition-Manufactured AlSi10Mg via Crystal Plasticity Finite Element Method

**DOI:** 10.3390/ma18102285

**Published:** 2025-05-14

**Authors:** Wang Zhang, Jianhua Liu, Yanming Xing, Xiaohui Ao, Ruoxian Yang, Chunguang Yang, Jintao Tan

**Affiliations:** 1 School of Mechanical Engineering, Beijing Institute of Technology, Beijing 100081, China; z_w_0112@163.com (W.Z.); jeffliu@bit.edu.cn (J.L.); 3220230321@bit.edu.cn (J.T.); 2 State Key Laboratory of Special Vehicle Design and Manufacturing Integration Technology, Inner Mongolia 014030, China; su_phoenix@163.com (Y.X.); chunguang33@126.com (C.Y.); 3 Hebei Key Laboratory of Intelligent assembly and Detection Technology, Tangshan 063015, China; yruoxian@163.com

**Keywords:** pore defects, stress concentration, crystal plasticity finite element, Voronoi model, LMD

## Abstract

Compared with powder metallurgy, centrifugal casting, jet molding, and other technologies, Laser Melting Deposition (LMD) stands out as an advanced additive manufacturing technology that provides substantial advantages in the melt forming of functional gradient materials and composites. However, when high-temperature and high-speed laser energy is applied, the resulting materials are susceptible to porosity, which restricts their extensive use in fatigue-sensitive applications such as turbine engine blades, engine connecting rods, gears, and suspension system components. Since fatigue cracks generally originate near pore defects or at stress concentration points, it is crucial to investigate evaluation methods for pore defects and stress concentration in LMD applications. This study examines the effect of pore defects on stress concentration in LMD-manufactured AlSi10Mg using the crystal plasticity finite element method and proposes a stress concentration coefficient characterization approach that considers pore size, morphology, and location. The simulation results indicate a competitive mechanism between pores and grains, where the larger entity dominates. Regarding the influence of aspect ratio on stress concentration, as the aspect ratio decreases along the stress direction, the stress concentration increases significantly. When pores are just emerging from the surface (s/r = 1), the stress concentration caused by the pore reaches its maximum, posing the highest risk of material failure. To assess the extent to which the aspect ratio, position, and size of pores affect stress concentration, a statistical correlation analysis of these variables was conducted.

## 1. Introduction

Additive manufacturing (AM), which is directly guided by 3D model data, is rapidly advancing as a promising production method across various industries. This technology provides substantial advantages, such as the capability to fabricate complex geometries and significantly reduce lead times. Laser Melting Deposition (LMD) is a metal printing technique that enables the production of components using powders with varying compositions through directed energy deposition. By constructing components layer by layer, LMD can achieve parts with nearly 100% density, thereby delivering performance that equals or even surpasses that of castings and forgings. Additionally, LMD technology can be employed to manufacture gradient materials and composite parts, enhancing the functional versatility of components and meeting specific requirements in biomedical [1], aerospace [2,3,4], and vehicle engineering [5] applications.

Research into the formation mechanisms, equipment, technology, and software systems for parts manufactured using Laser Metal Deposition (LMD) technology has reached a high level of maturity. Various process optimization methods, including experimental approaches [6], simulations [7], and deep learning techniques [8], have been employed to produce parts with superior mechanical properties. However, under the influence of high-speed and high-energy lasers, pore defects inevitably form during the manufacturing process [9]. This issue is particularly pronounced when fabricating gradient materials via the LMD process, as physical properties such as thermal conductivity, thermal expansion coefficient, and the solid–liquid phase line continuously vary with changes in powder composition. As a result, the solidification behavior of the melt pool becomes less stable, increasing the likelihood of pore defect formation and leading to a fatigue life that is significantly lower than that of forged components. Moreover, variations in the size, shape, and distribution of these pores substantially affect the consistency of fatigue performance in parts produced under identical processing conditions [10]. The unique characteristics of these pores pose challenges for detection, potentially causing unexpected and catastrophic engineering failures and resulting in significant economic losses.

Given that fatigue cracks typically nucleate at stress concentration locations such as pores and inclusions, these defects substantially impair the fatigue life of additive manufacturing (AM) components. They are the primary factors responsible for the inferior fatigue performance of metal AM parts compared to their wrought counterparts [11]. Specifically, pore defects represent the most critical factor inducing local stress concentrations in metal components fabricated via additive manufacturing. These defects frequently act as initiation sites for fatigue cracks, significantly influencing fatigue damage accumulation, fatigue life, and crack initiation locations in additively manufactured metal materials [12]. As discontinuities on or within the material surface, pores facilitate crack initiation and propagation under impact or cyclic loading conditions [13,14]. For instance, in aircraft engine turbine blades, compared with fully dense materials, pores compromise the continuity of nickel-based superalloys, rendering the material more susceptible to brittle fracture and thereby diminishing its toughness and fatigue resistance [15].

To date, no effective method has been developed to completely eliminate these defects [16]. Consequently, numerous researchers have investigated the relationship between pore defects and fatigue strength. Several well-known models have been proposed, including the Murakami method [17], the Kitagawa–Takahashi fatigue diagram, and the Extreme Value Statistics (EVS) approach [18]. Hu et al. [19] employed EBSD and CT technology to quantify porosity, subsequently combining the EVS and Murakami models to evaluate fatigue strength based on defect quantity. They further established an extended Kitagawa–Takahashi fatigue diagram within the framework of defect-tolerant design. Sanaei et al. [20] utilized EVS to approximate initial defect size and Murakami’s method to determine equivalent defect size, providing essential characteristics for selecting appropriate crack growth equations to describe crack propagation regimes. Liang [21] applied the Murakami approach to analyze the relationship between fatigue strength and defect size, as well as the fatigue strength ratio under different loading modes. The study revealed that multiple clustered defects act synergistically as a single large defect in initiating fatigue cracks. In situ fatigue tests monitored by X-ray synchrotron tomography demonstrated that surface cracking initiated at defects up to ten times smaller than internal defects systematically dominated the internal nucleation and propagation of cracks [22].

Both fractography and CT analysis reveal that the earliest crack nucleation occurs in regions with the highest stress concentrations [23]. Consequently, evaluating stress concentration provides a reliable indicator of a material’s propensity for early defect nucleation [24,25]. Initial defects within laser metal deposition (LMD) specimens, combined with machining-induced surface defects, result in stress concentrations that further promote crack initiation during vibration fatigue testing [26]. To quantitatively analyze the influence of pore defects on stress concentration from an evolutionary mechanism perspective and to provide a reference for assessing material fatigue performance, the finite element method has been extensively employed to study the stress behavior associated with various microstructural defects. Borbély [27] investigated the stress and strain fields around spherical cavities located at varying distances beneath the surface of a tensile-stressed material using finite element modeling. The findings indicate that under cyclic loading conditions, strain localization near the surface of a cavity just beneath the material surface can be severe enough to initiate subsurface fatigue cracks. Conversely, when a pore is located more than one diameter away from the free surface, its effect on the stress concentration factor (Kt) becomes negligible [28]. Xu et al. [29] analyzed the variation of the stress field around a pore as a function of depth within a linear elastic solid using finite element modeling. Their results demonstrate that pore depth significantly influences the stress field around surface-intersecting pores, with the maximum stress increasing sharply when the pore intersects the surface at its top. Zhao et al. [30] showed that as pore size increases from 10 µm to 50 µm and the distance between the pore and the material surface decreases from 50 µm to 10 µm, stress concentration becomes increasingly severe. A model describing Kt as a function of pore characteristics (pore diameter and distance from the surface) was developed based on elasto–plastic behavior and validated with a prediction error of up to 3% [31].

Although the boundary stresses remain within the elastic limit of the material, stress concentrations around pores result in the formation of localized plastic zones, thereby accelerating damage accumulation within the material. The significance of pore-induced stress concentration is reflected not only in the stress concentration factor but also in the spatial extent of the affected regions. Given the considerable complexity involved in identifying fatigue crack initiation and predicting fatigue life, establishing a universal mechanism or rule remains challenging. In this study, we developed a crystal plasticity finite element model to systematically investigate the mechanisms underlying stress formation and the distribution patterns of pores with varying characteristics. Furthermore, we analyzed the influence of different pore characteristics on stress concentration and proposed a comprehensive characterization methodology for pore defects associated with fatigue damage induced by stress concentration.

## 2. Theory Model

In classical crystal plasticity theory, the crystal structure, hardening modulus, and crystal orientation are taken into account. The total deformation of crystalline materials comprises both elastic deformation and plastic deformation [32,33]. Consequently, the total crystal deformation gradient *F* can be represented as

(1) $$F=F_{e}\cdot F_{p}$$

where *F_e_* is the elastic deformation gradient caused by lattice distortion and the rigid body rotation, *F_p_*, is the plastic deformation gradient related to the deformation mechanism, such as slip, twin, phase change, etc. An AlSi10Mg alloy consists of a large number of primary crystal α-Al phases with a surface-centered cubic structure and fine particle eutectic Si. The dislocation slip has 12 slip systems with four sliding surfaces of {111} and three slip directions of <110>, and its plastic deformation is mainly caused by dislocation slip, especially through the octahedral {111}<110> slip system, so the plastic deformation gradient $F_{p}$ is mainly considered. The plastic velocity gradient can be expressed as the sum of the shear strain rates of all slip systems

(2) $$L_{p}=\sum_{\alpha =1}^{N}{\dot{\gamma }}_{\alpha }S_{\alpha }⊗m_{\alpha }$$

where *N* is the number of slip systems, ${\dot{\gamma }}_{\alpha }$ is the plastic slip shear rate, $S_{\alpha }$ and $m_{\alpha }$ are the unit vectors in the slip direction and the normal vectors of the slip system *α,* respectively. The shear strain rate ${\dot{\gamma }}_{\alpha }$ is given as

(3) $${\dot{\gamma }}_{\alpha }={\dot{\gamma }}_{0}{\left(\frac{\tau_{\alpha }}{g_{\alpha }}\right)}^{n}sgn\left(\tau_{\alpha }\right)$$

where ${\dot{\gamma }}_{0}$ is the initial shear strain rate, $g_{\alpha }$ is the strain-hardening function of the slip system *α*, $\tau_{\alpha }$ is the cutting stress, and n the sensitive coefficient of strain rate.

The hardening function $g_{\alpha }$ is assumed to be related to the total slip *γ* in all slip systems

(4) $$g_{\alpha }=g_{\alpha }\left(\gamma \right)$$

(5) $$\gamma =\sum_{\alpha =1}^{N}\int_{0}^{t}\left|{\dot{\gamma }}_{\alpha }\right|dt$$

The strain hardening law of a single crystal is given as

(6) $${\dot{g}}_{\alpha }=\sum_{\beta =1}^{N}h_{\alpha \beta }\left|{\dot{\gamma }}_{\beta }\right|$$

In different slip systems where α ≠ β, $h_{\alpha \beta }$ is a hardening modulus, in the same slip systems where α =β, $h_{\alpha \alpha }$ is a self-hardening function

(7) $$h_{\alpha \beta }=qh+\left(1-q\right)h\delta_{\alpha \beta }$$

(8) $$h\left(\gamma \right)=h_{0}{\mathrm{sech}}^{2}\left|\frac{h_{0}\gamma }{\tau_{s}-\tau_{0}}\right|$$

where, q is the ratio of self-hardening effect to latent hardening effect, and the value is 1 or 1.4. $h_{0}$ is the initial hardening rate, $\tau_{0}$ is the yield, and $\tau_{s}$ is the saturated flow stress.

## 3. Modeling Based on the Experimental Data

### 3.1. Voronoi Multicrystal Model

**Figure 1:**
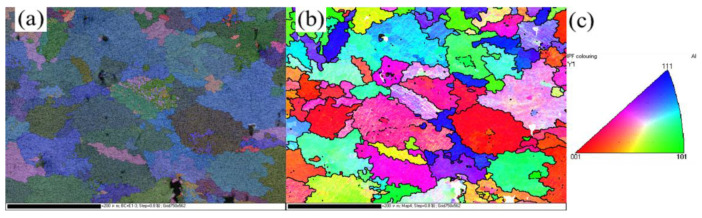
(**a**) EDS, (**b**) EBSD and (**c**) IPF of LMD-manufactured AlSi10Mg.

**Figure 2:**
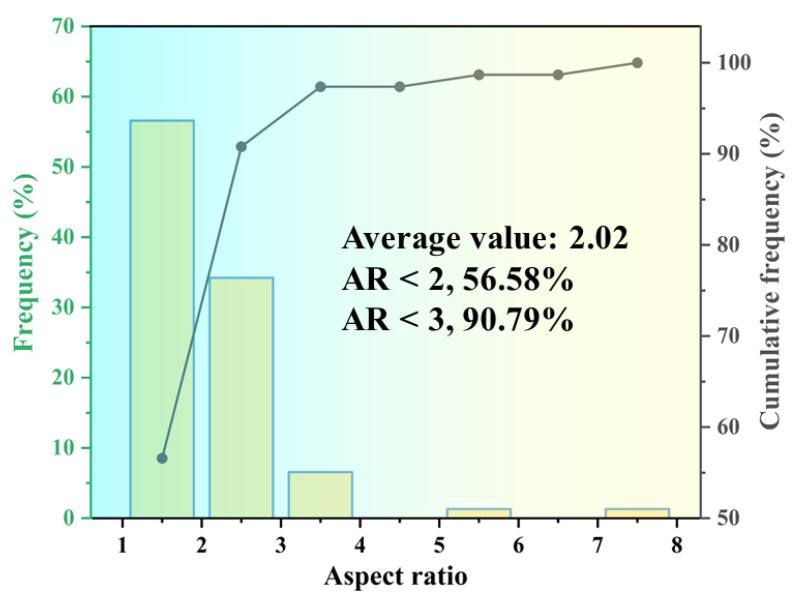
The aspect ratio of the AlSi10Mg grains was quantified.

The material investigated in this study is LMD-manufactured AlSi10Mg. Specimens were fabricated using three distinct laser power settings: 1500 W, 1800 W, and 2100 W. Additional process parameters included a scanning speed of 20 mm/s, a powder feed rate of 2.5 g/min, an overlap ratio of 56%, a layer thickness of 0.9 mm, and a laser spot size of 2.5 mm. Prior to each deposition cycle, the chambers were meticulously cleaned to prevent cross-contamination. Electron backscatter diffraction (EBSD) was utilized to analyze the grain morphology and orientation by MIRA3(TESCAN, Brno, Czech Republic) as depicted in Figure 1. The EBSD results indicated nearly equiaxed grains with an average grain size ranging from 50 to 60 μm. No significant texture was observed in the microstructure. The aspect ratio of the AlSi10Mg grains was quantified, as presented in Figure 2. Specifically, grains with an aspect ratio below 3 accounted for 90.79%, while those below 2 constituted 56.58%.

**Figure 3:**
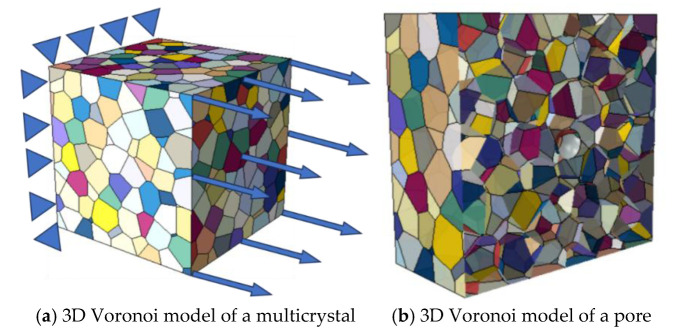
Three-dimensional Voronoi model of a (**a**) multicrystal and (**b**) pore.

Compared with the homogeneous finite element model, the crystal plasticity finite element model incorporates interaction behaviors such as shear and sliding between individual grains at the microscopic level. This approach provides a more accurate representation of the cumulative influence of material defects and microstructure on mechanical properties. The Voronoi multicrystal model for LMD-manufactured AlSi10Mg alloy crystals was generated using Python 3.9 code based on the Voronoi tessellation algorithm, as depicted in Figure 3a. The computational domain had dimensions of 500 × 500 × 500 μm³. Given that pore structures are critical factors influencing the fatigue properties of materials, this study developed a Voronoi-based pore model, as shown in Figure 3b, to further examine the impact of pores on stress concentration in AlSi10Mg alloys.

**Figure 4:**
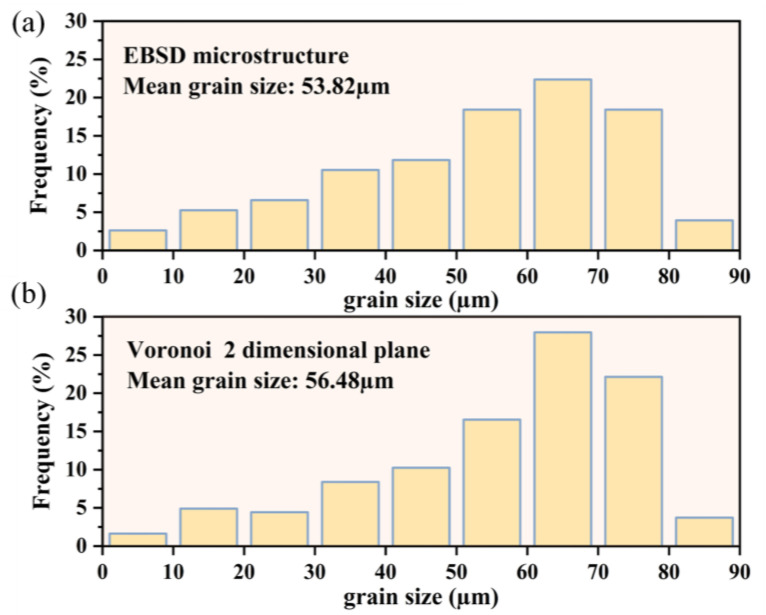
Grain distribution (**a**) measured by EBSD and (**b**) calculated by the Voronoi model.

According to the grain distribution measured by EBSD, as shown in Figure 4a, the average and maximum grain size of the Voronoi model was set within the range of 50 to 60 μm and 80 to 90 μm. The grain distribution of the Voronoi model closely resembled the experimental data, as illustrated in Figure 4b. The random initial grain orientations and the Euler angle data for these grain morphologies were generated through a user-defined material mechanical behavior (UMAT). This procedure was implemented using Python code, and the relevant parameters are detailed in Table 1. The model comprised 600 grains with a grid type of C3D4H, divided into 71,694 cells and 12,844 nodes.

### 3.2. Crystal Plasticity Finite Element Method

**Figure 5:**
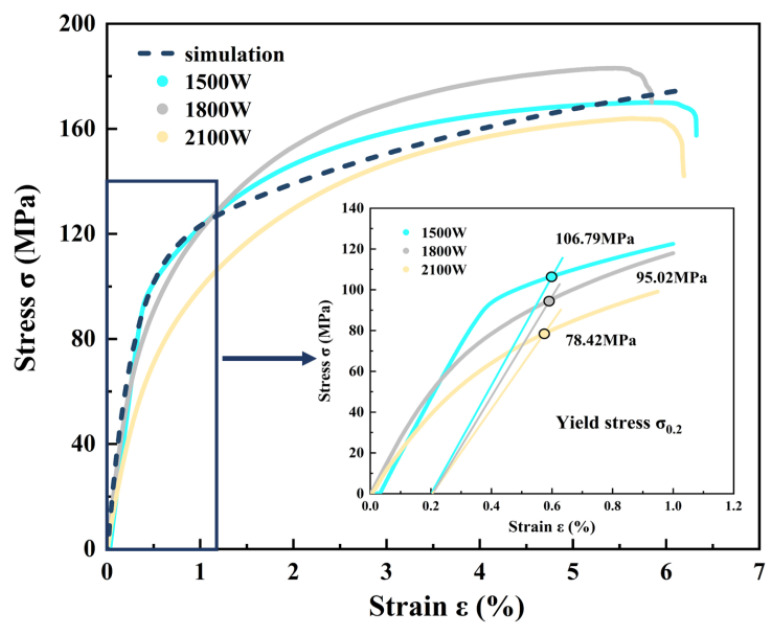
Tensile stress and strain curves of samples prepared with three different powers.

In accordance with the ASTM E466-21 standard, tensile specimens were prepared and subjected to tensile testing. The tests were conducted at room temperature using the UTM4000 series electronic universal testing machine, which features a maximum testing force range of 100 N to 30 kN. Specimens fabricated under three distinct power settings were subjected to axial tension at a strain rate of 0.001 s⁻¹. Each test was repeated three times to ensure the precision and reliability of the experimental data, yielding the tensile stress–strain curves presented in Figure 5.

**Figure 6:**
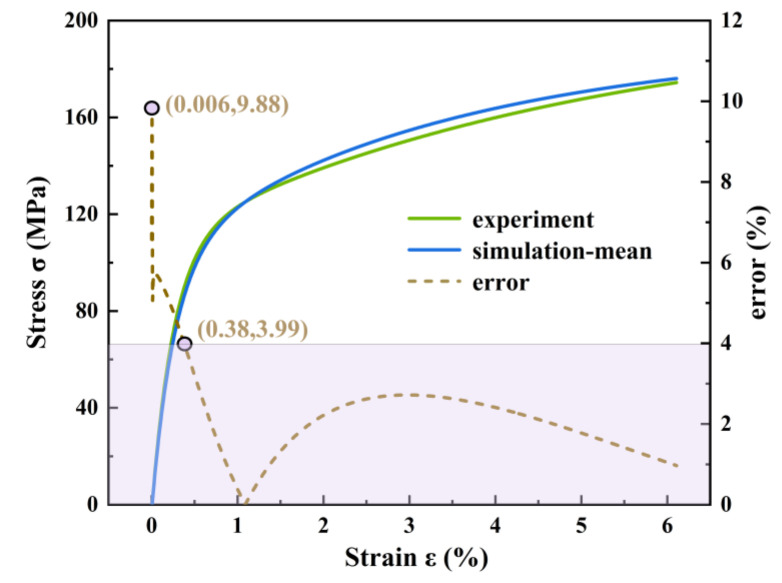
Average stress–strain error curve obtained from multiple simulations.

The parameters of the crystal plasticity model were calibrated by comparing them with the tensile stress–strain curves of the AlSi10Mg alloy. For crystal plasticity models with an identical average grain size, each simulation generated 600 grains with varying orientations and morphologies. This process produced a uniaxial tensile stress–strain curve up to a strain value of 6%. The averaged stress–strain curves obtained from multiple simulations are presented in Figure 6. Upon comparison with the experimental and simulation results, the stress–strain curve simulated by the crystal plasticity model exhibits close agreement with the experimental data. The relevant physical parameters of AlSi10Mg are summarized in Table 1.

**Figure 7:**
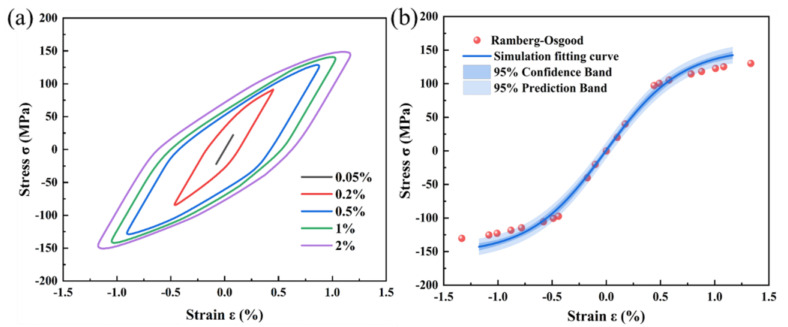
(**a**) Stress–strain hysteresis curve, (**b**) stress–strain curve of a stable cycle.

Low-cycle fatigue simulations were performed using the crystal plasticity UMAT subroutine and the Voronoi model. Various strain amplitudes were applied to one boundary of the Voronoi model to simulate stress–strain curves, and the recorded stress–strain hysteresis loops are presented in Figure 7a. The relationship between the vertices of steady-state hysteresis loops reflects the stress amplitude response under different strain amplitudes, as depicted in Figure 7b. The classical cyclic stress–strain curve is mathematically described by the Ramberg–Osgood relationship as

(9) $$\varepsilon_{a}=\varepsilon_{ea}+\varepsilon_{pa}=\frac{\sigma_{a}}{E}+{\left(\frac{\sigma_{a}}{K}\right)}^{\frac{1}{n}}$$

where ε_ea_ is the cyclic elastic strain, ε_pa_ is the cyclic plastic strain under cyclic loading, *E* is the elastic modulus, *K* is the cyclic strength coefficient, and n is the cyclic strain hardening exponent. These parameters were determined through curve fitting, yielding results of *E* = 20.73 GPa, *K* = 180 MPa, and *n* = 0.06. The simulation results show good agreement with the Ramberg–Osgood equation within the 95% confidence interval, verifying that the established crystal plasticity finite element model exhibits favorable reliability.

## 4. Results and Discussion

For components produced through additive manufacturing, crack initiation usually occurs at the most critical defect and is influenced by the stress state at that location. Murakami proposed a fundamental formula for the defect stress intensity factor, considering both the location and area of the defect, as follows:

(10) $$K=\sigma \cdot Y\cdot {\left[\pi \cdot {\left(area\right)}^{1/2}\right]}^{1/2}$$

where σ is the force applied, Y is the geometric correction factor used to characterize the defect locations (for the surface defects, Y  = 0.65; for the near-surface or internal defects, Y  = 0.5). However, Equation 9 cannot reflect the effect of various factors on stress concentration. The relative stress strength factor takes into account the location, size, and morphology of defects, as well as their interaction with surface defects and the surrounding material. It also considers the effects of uneven stress distribution resulting from geometric variations in the component. This factor is defined as follows:

(11) $$K_{relative}=f\left(K_{t}\cdot \sigma_{x}{\left(\pi \sqrt{area}\right)}^{1/2}\right)$$

where $\sigma_{x}$ is the local stress considering the inhomogeneity of the stress distribution in the material and $K_{t}$ is the stress concentration factor reflecting the degree of stress concentration caused by the location and morphology of the defect and the interaction between defects and material surfaces or adjacent defects. $K_{t}=\sigma_{x}/\sigma_{0}$, where $\sigma_{max}$ is the peak stress and $\sigma_{0}$ is the far-field stress. In fact, the stress concentration factor is positively correlated with the relative stress strength factor, allowing for the evaluation of a material’s fatigue performance through the comparison of stress concentration factors. This paper summarizes the effects of pore size ($K_{ts}$), pore location ($K_{tl}$), and pore aspect ratio ($K_{ta}$) on stress concentration. Additionally, it discusses the mechanisms by which pore characteristics and crystal structure influence fatigue life.

### 4.1. The Effect of Pore Size

**Figure 8:**
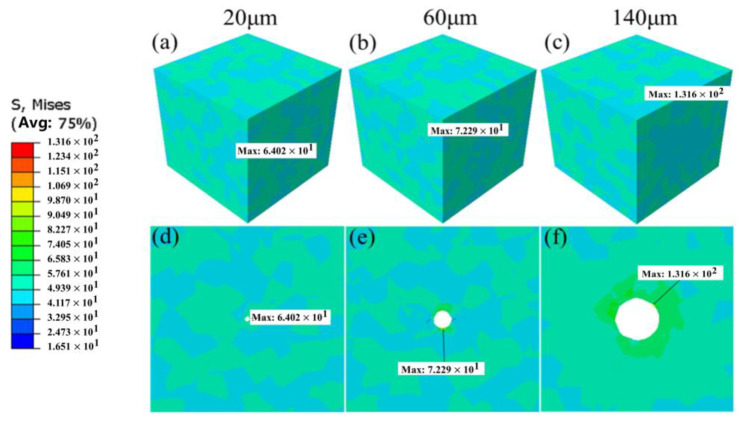
Distribution of von Mises stress with pore sizes of 20 μm, 60 μm, and 140 μm, respectively. (**a**–**c**) Three-dimensional model; (**d**–**f**) two-dimensional cross section.

Pore size has a substantial impact on fatigue life, and the Murakami parameter is frequently employed as one of the evaluation indices for this defect level. Figure 8 illustrates the distribution of von Mises stress for pore sizes of 20 μm, 60 μm, and 140 μm, respectively. It can be observed that when the pore size is 20 μm, the maximum von Mises stress and cumulative plastic slip occur at the grain boundaries surrounding the pore. At this stage, stress concentration is predominantly governed by grain orientation, and the combined action of all active slip systems results in significant cumulative plastic slip at this location. When the pore diameter increases to 60 μm, the highest von Mises stress is located at the pore edge, while the peak cumulative plastic slip occurs at the grain boundaries surrounding the pore, influenced by the synergistic effects of grain orientation and pore presence on stress concentration. When the pore size expands to 140 μm, the peak von Mises stress and cumulative plastic slip are concentrated at the pore edge, with the stress distribution exhibiting a tendency to spread around the pore region. These findings indicate that pore size significantly affects stress concentration and cumulative plastic slip during the process.

**Figure 9:**
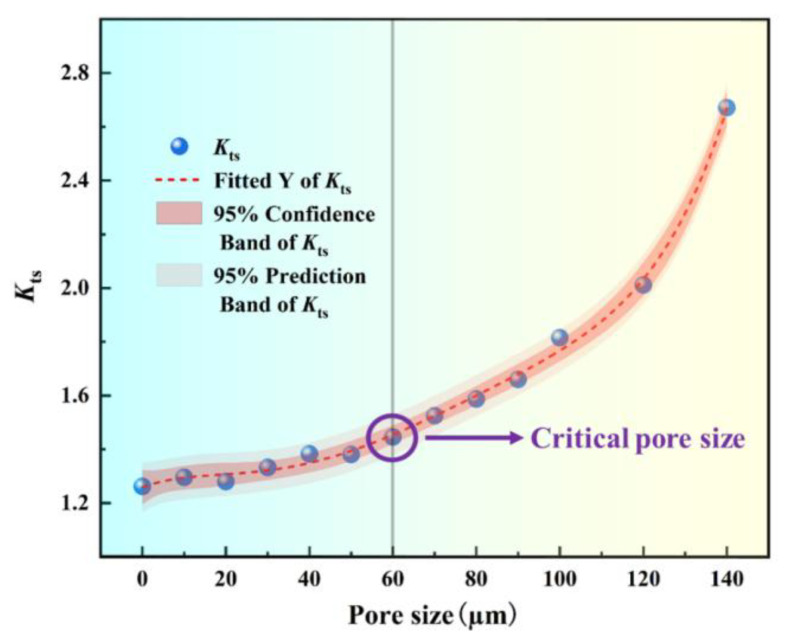
Variation of the stress concentration coefficient $K_{ts}$ with the pore size from 0 to 140 μm.

The variation of the stress concentration coefficient with pore size, ranging from 0 to 140 μm, is depicted in Figure 9. It can be observed that the stress concentration coefficient is positively correlated with pore size, and as the pore size increases, the stress concentration gradually rises. Specifically, when the pore size increases from below 60 μm, the stress concentration coefficient progressively increases from approximately 1.2 to 1.5. However, as the pore size exceeds 60 μm, the stress concentration coefficient rapidly escalates, rising from 1.5 at 60 μm to 2.6 at 140 μm, corresponding to an increase rate of approximately 73%. Notably, 60 μm represents the average grain size. Therefore, it can be concluded that when the pore size is smaller than the average grain size, grain orientation plays a dominant role in stress concentration. Conversely, when the pore size exceeds the average grain size, the influence of the pore on stress concentration becomes critical and significantly intensifies.

From the aforementioned comparison, it can be observed that both pore size and grain size influence stress distribution, induce stress concentration, and subsequently determine the initiation site of fatigue cracks. Consequently, a competitive mechanism exists between pores and grains. When the pore size is smaller than the average grain size, stress concentration is predominantly governed by grain size and occurs at the junctions of multiple grains, with microstructure becoming the critical factor controlling material fatigue strength. When the pore size is comparable to the average grain size, stress concentration is jointly dominated by pore presence and grain orientation, with the stress concentration point located at the polycrystalline boundary surrounding the pore. When the pore size exceeds the average grain size, stress concentration occurs at the pore edge. As pore size increases, the stress concentration coefficient rises rapidly. These findings suggest that when the pore size surpasses the average grain size, stress concentration is primarily dictated by the pore.

**Figure 10:**
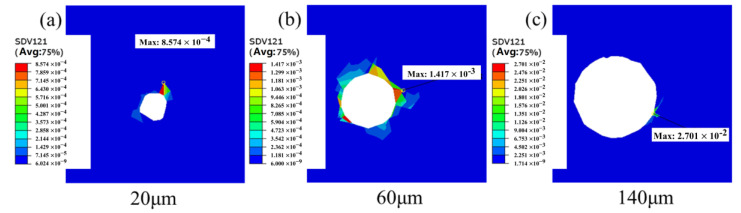
Cumulative slip at pore diameter (**a**) D = 20 μm, (**b**) D = 60 μm and (**c**) D = 140 μm.

In this study, the concept of “critical damage size” was formally defined, indicating that pores begin to significantly influence fatigue damage only after reaching this specific size. The “critical damage size” is primarily determined by the mechanisms and locations of stress concentration, in conjunction with the sharp transitions in the Kt value and the accumulated slip amount. We analyzed the stress concentration and accumulated plastic slip for three pore sizes: 20 μm, 60 μm, and 140 μm, as illustrated in Figure 10. As the pore size increases, the maximum values of von Mises stress and accumulated plastic slip progressively shift from the grain boundaries surrounding the pore to the pore edge. When the pore size reaches 60 μm (approximately equal to the average grain size), the highest von Mises stress occurs at the pore edge, while the peak accumulated plastic slip remains at the grain boundaries around the pore. This phenomenon arises under the combined influence of grain orientation and pore presence on stress concentration, representing the “transition state” in the competition between pore and grain size. Consequently, the “critical damage size” for material pores in this study is determined to be within the range of 50–60 μm. For pores smaller than 20 μm, the induced stress concentration effect is negligible. Furthermore, it is challenging to detect pores below 20 μm using CT scanning, and a portion of these may be appropriately disregarded without significant statistical impact.

### 4.2. The Effect of Aspect Ratio

**Figure 11:**
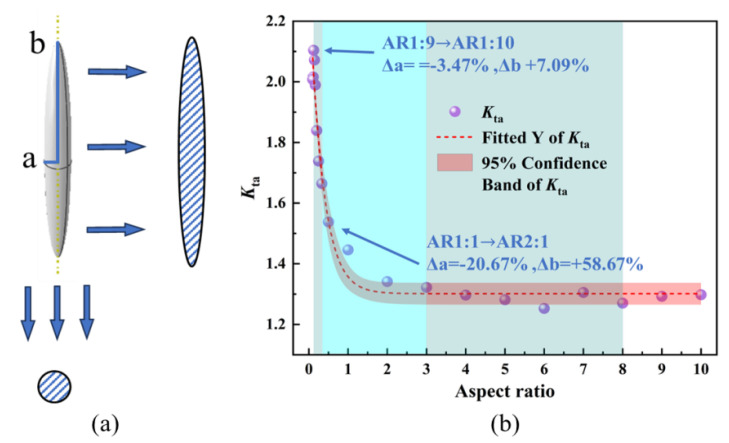
(**a**) Definition of aspect ratio and (**b**) variation of the stress concentration coefficient with the pore AR from 1:10 to 10:1.

**Figure 12:**
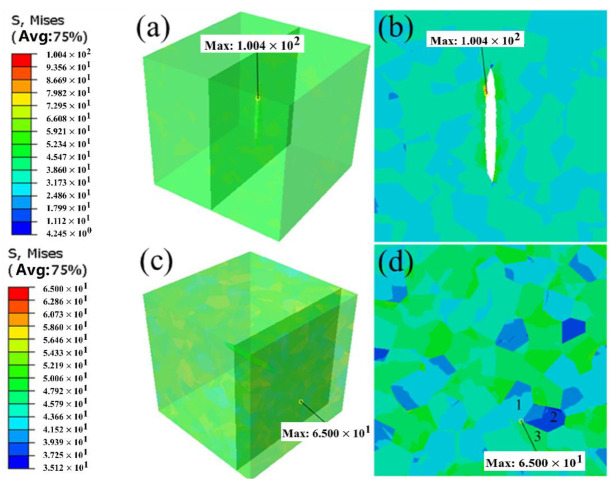
Simulation results of AR at two “extreme” ratios of (**a**,**b**) AR = 1:10 and (**c**,**d**) AR = 10:1.

Pore morphology is also a critical factor influencing the stress concentration coefficient. The pore aspect ratio (AR) is commonly employed to quantify the impact of pore morphology on stress concentration. This method simplifies complex, distorted pores or defects into idealized geometries with straightforward morphologies, thereby enabling a deeper understanding of micromechanical processes within microstructures. The aspect ratio is defined as the ratio of the long axis *b* of the pore to its short axis *a*, as depicted in Figure 11a. Figure 12a presents the simulation results for two “extreme” aspect ratios: 1:10 and 10:1. From Figure 12b, it can be observed that the stress concentration points are predominantly located at the edges of the elliptical pore, and these findings align well with the relevant literature. In this case, the pore resembles a micro-crack, forming a large “fish-eye” crack under tensile loading. Due to the presence of two sharp tips, the induced stress concentration effect is highly pronounced. From Figure 12d, it can be seen that the maximum stress value does not occur around the pore but rather at the grain boundary junctions of multiple grains. This indicates that the influence of grain orientation on stress concentration outweighs that of pore morphology, highlighting the significant role of microstructure in determining stress distribution.

Figure 11b illustrates the variation of the stress concentration coefficient with the pore aspect ratio (AR) ranging from 1:10 to 10:1. By maintaining a constant pore volume and altering the dimensions of the long and short axes, it can be observed that when the aspect ratio is less than 1, the stress concentration increases as the aspect ratio decreases. As the aspect ratio increases from 1:1 to 10:1, the maximum stress value decreases until stabilization occurs, accompanied by a corresponding change in stress distribution. Notably, during this process, the projection surface remains circular, effectively reducing the radius of the circular pore. Consequently, the influence pattern of this process closely resembles the scenario of decreasing pore size from 60 μm to 10 μm. Since the projected diameter in this case remains consistently smaller than the critical damage size of 60 μm, the stress concentration is determined by grains 1, 2, and 3 rather than by the pore itself. This observation further validates the rationality of the competition mechanism between pore and grain size. When the AR decreases below 1:1, due to the large degree of projected area variation, this results in a fast increase in the $K_{\mathrm{ta}}$. However, in the “extreme” case of 1:10, the size of the projected area can only be slightly changed by increasing the AR, and the change of $K_{\mathrm{ta}}$ value tends to saturation, indicating that the degree of size change of the projected area affects the rate of change of $K_{\mathrm{ta}}$.

### 4.3. The Effect of Pore Location

**Figure 13:**
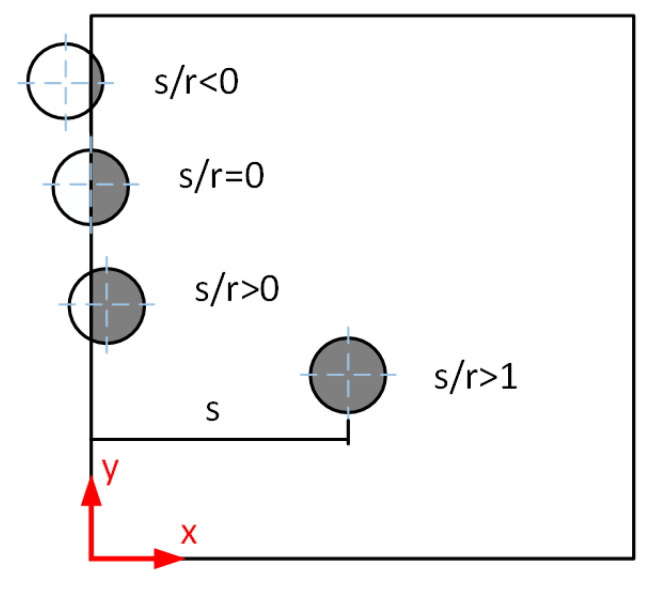
Location of the pore relative to the material surface.

Figure 13 shows the location of the pore relative to the material surface. The pore locations were characterized by the relationship s/r between the pore radius r and the distance s from the pore center to the nearest surface. s/r < 1 shows that the pore is a surface defect and s/r > 1 indicates that the pore has translated into a material internal defect.

**Figure 14:**
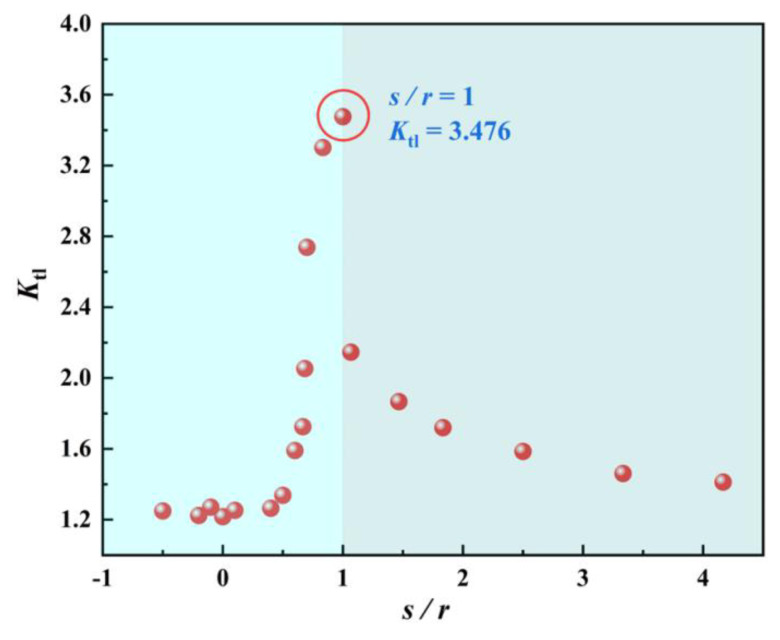
Variation of the stress concentration coefficient with the pore location.

Figure 14 shows the variation of the stress concentration coefficient with respect to the pore location. When s/r < 0.5, the pore location had a minor effect on $K_{\mathrm{tl}}$, whereas when s/r > 0.5, with increasing s/r, the stress concentration coefficient increases rapidly to the maximum value of 3.476 at s/r = 1. The effect of the pore on the stress concentration coefficient rapidly decreases to 1.6 at s/r = 2.5 and then slows down. This suggests that greater stress concentration is generated in the pores close to the material surface, while the internal pores affect the stress concentration to a smaller extent.

### 4.4. Comprehensive Influence

**Figure 15:**
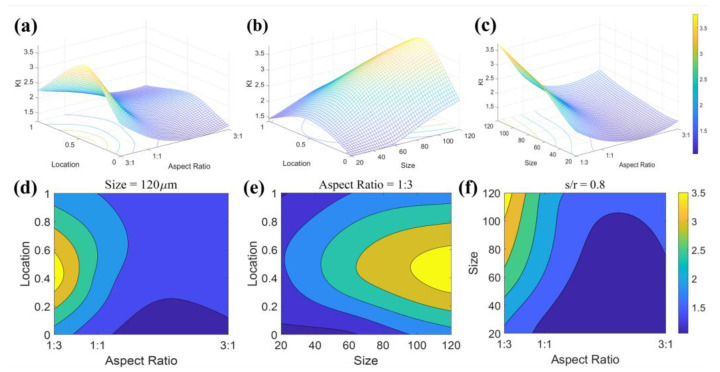
Interactive effects of pore size = 120 μm, aspect ratio = 1:3, and s/r = 0.8 on the stress concentration coefficient $K_{t}$ (**a**–**c**) Three-dimensional surface, (**d**–**f**) two-dimensional projection.

To study the comprehensive effects of pore size, aspect ratio, and location on stress concentration, the stress concentration coefficients for various characteristic pores are calculated using the crystal plasticity finite element model established earlier. Figure 13 illustrates the interactive effects of pore size = 120 μm, aspect ratio = 1:3, and s/r = 0.8 on the stress concentration coefficient $K_{t}$. In Figure 15a, when the pore aspect ratio exceeds 1:1, the stress concentration coefficient $K_{t}$ is smaller, and the location of the pore has a minimal effect. As the pore aspect ratio decreases, $K_{t}$ increases rapidly, and the influence of pore location also becomes more pronounced. In Figure 15b, when the aspect ratio is 1:3, the pore size significantly affects $K_{t}$. As the pore size increases, especially near the material surface, $K_{t}$ increases at a faster rate. In Figure 15c, for pores located at the material surface, when the size is smaller than the grain size and the aspect ratio is greater than 1:1, no significant stress concentration is observed.

**Figure 16:**
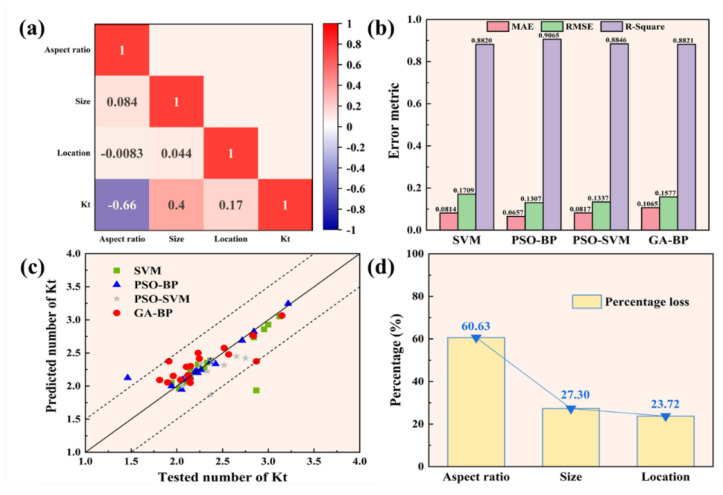
(**a**) PCC matrix illustrating the relationship between various pore features and the stress concentration coefficient $K_{t}$; (**b**) performance comparison among different machine learning algorithms; (**c**) comparison of machine learning predictions of $K_{t}$ with the dataset values of $K_{t}$; (**d**) impact of different pore features on the performance of the machine learning model.

To quantitatively predict the impact of various pore features on stress concentration, we evaluated the predictive performance of four distinct machine learning algorithms. Figure 16a presents the Pearson correlation coefficient (PCC) matrix for three key pore features: size, aspect ratio, and location. All variables are mutually independent and collectively influence the output. Positive covariance values indicate a positive correlation, whereas negative values indicate a negative correlation. The results reveal that the pore aspect ratio has the most substantial effect on stress concentration, followed by pore size and pore location. The prediction accuracy based on different machine learning algorithms is illustrated in Figure 16b. Among all algorithms, the PSO-BP algorithm demonstrated superior performance, achieving the highest goodness-of-fit while exhibiting minimal root mean square error (RMSE) and mean absolute error (MAE). Figure 16c further depicts the relationship between the $K_{t}$ values predicted by various machine learning algorithms and the actual dataset $K_{t}$ values. Excluding an outlier with a predicted value exceeding the actual value, the PSO-BP algorithm exhibits a high goodness of fit, with the overall distribution closely aligned along the diagonal. To further quantify the impact of each pore feature, we trained models using different combinations of pore features via the PSO-BP algorithm, with the results displayed in Figure 14d. When disregarding the effects of aspect ratio, size, and location, the goodness of fit decreased by 60.63%, 27.3%, and 23.72%, respectively. These findings confirm that the pore aspect ratio has the most significant influence on stress concentration, followed by pore size and pore location.

## 5. Conclusions

Stress concentration is a critical factor in the initiation of fatigue cracks, particularly as stomatal defects-induced stress concentration significantly reduces fatigue life. In this study, the stress concentration coefficient for pores in LMD-manufactured AlSi10Mg was investigated using the Voronoi crystal model and the crystal plasticity finite element method. The influence mechanisms and laws of pore size, location, and aspect ratio on the stress concentration coefficient were analyzed systematically, leading to the following conclusions:

(1) A competitive mechanism exists between pores and grains regarding size in determining stress concentration. The larger structure (pore or grain) governs stress concentration and cumulative plastic slip. When the pore size exceeds the “critical damage size,” the stress concentration effect is predominantly determined by the pore. For LMD-manufactured AlSi10Mg in this study, the critical damage size is within the range of 50–60 μm.

(2) The influence of pore morphology on stress concentration is primarily attributed to the long axis of the pore and the angle of the applied force. For pores with the same area, a longer equivalent pore length perpendicular to the direction of the applied load results in greater stress concentration. Therefore, the presence of “sharp” geometric features should not be overlooked during the analysis of local plastic deformation processes; even when the aspect ratio exceeds 1, significant stress concentration can still occur.

(3) When the aspect ratio is less than 1, the stress concentration coefficient increases rapidly as the porosity ratio decreases. Eventually, the aspect ratio surpasses the effects of pore size and position, becoming the dominant factor influencing stress concentration. However, as the pore aspect ratio decreases, the stress concentration coefficient diminishes and at smaller aspect ratios the sensitivity of pore size and position to the stress concentration coefficient reduces.

## Figures and Tables

**Table 1:** Related physical parameters of AlSi10Mg.

C_11_/MPa	C_12_/MPa	C_44_/MPa	*h*_0_/MPa	*τ*_s_/MPa	*τ*_0_/MPa	*n*	$\gamma_{0}$	*q*
45,366	25,673	12,044	145	45.9	27.9	20	0.001	1

## Data Availability

The data in this paper are all obtained from experiments. All data generated during this study are included in this manuscript.
